# Comparing thermal strain in outdoor maintenance and indoor service workers in the mining industry during summer

**DOI:** 10.1371/journal.pone.0292436

**Published:** 2023-10-05

**Authors:** Sarah M. Taggart, Olivier Girard, Grant J. Landers, Ullrich K. H. Ecker, Karen E. Wallman

**Affiliations:** 1 School of Human Sciences, The University of Western Australia, Crawley, WA, Australia; 2 School of Psychological Science, The University of Western Australia, Crawley, WA, Australia; Tokat Gaziosmanpasa University Tasliciftlik Campus: Tokat Gaziosmanpasa Universitesi, TURKEY

## Abstract

While working in the heat is a common practice within the Australian mining industry, it can lead to adverse effects on cognitive function, productivity, and physical health. This study aimed to compare the thermal strain experienced by maintenance workers and service workers in the mining industry during summer. Psycho-physiological parameters, manual dexterity, and cognitive function were assessed in maintenance workers (n = 12) and service workers (n = 12) employed at mine site villages in north-west Australia. Maintenance workers had the freedom to self-select their work intensity and predominantly worked outdoors (33.9±4.2°C, 38±18% RH), whereas service workers had to work to a fixed schedule and worked intermittently indoors (∼64% of work shift; 29.5±3.4°C, 48±8% RH) and outdoors (∼36%; 35.4±4.6°C, 47±21% RH). All workers underwent assessment at the beginning (day 2/3), middle (day 7/8), and end of their swing (day 13/14), at various time points throughout their 11–12 h shift. Service workers completed more steps (11282±1794 *vs*. 7774±2821; p<0.001), experienced a higher heart rate (p = 0.049) and reported higher ratings of perceived exertion (p<0.001), thermal discomfort (p<0.001), thermal sensation (p<0.001), and fatigue (p<0.012) compared to maintenance workers. Urinary specific gravity values were higher (less hydrated) in service workers (1.024±0.007) compared to maintenance workers (1.018±0.006; p = 0.007), with USG being overall higher post- compared to pre-shift (1.022±0.008 *vs*. 1.020±0.006; p<0.05). Core temperature, working memory capacity, processing speed and manual dexterity did not differ between occupations. Workers in hot environments who cannot self-select their work intensity should be educated about the importance of hydration before, during, and after their work-shifts and provided with more scheduled rest breaks during their shift. Employers should closely monitor workers for symptoms of heat illness, discomfort, and fatigue to ensure the health and safety of the workers.

## Introduction

Working in the heat for extended periods can cause physiological strain, leading to adverse effects on productivity [1] and cognitive function [2], with dehydration likely to exacerbate these effects [3]. A study by Ioannou and colleagues [4] reported that workers exposed to solar radiation in the heat experienced higher skin temperatures and an increased risk of heat stress symptoms compared to those working without solar radiation. In a laboratory, participants exposed to solar radiation performed poorly on cognitive tasks related to attention and vigilance, compared to those exposed to similar thermal stress without sunlight [4]. In the mining industry, Hunt and colleagues [5] assessed 15 blast crew workers, who had a mean urinary specific gravity of 1.024 (indicating significant dehydration), and found that 73% of these workers reported at least one symptom related to heat illness. Hence, inadequate management of heat exposure could lead to occupational heat strain, potentially resulting in heat illness [6] and an increased risk of injury [7].

To address thermal strain and ensure the health and safety of staff, workplaces can implement behavioural thermoregulatory countermeasures. For example, workplaces can provide guidance to outdoor workers, encouraging them to seek or create shade when feasible during work hours [8]. Indoor workers can benefit from strategies such as the use of air coolers or fans [8]. Both indoor and outdoor workers facing hot conditions could also stay hydrated by drinking cold water, using ice packs, taking more frequent rest breaks, or even suspending work at certain temperatures [8, 9]. Importantly, performing manual work in the heat that requires sustained attention without adequate rest can lead to significant fatigue among workers [10]. To counteract this, workplaces, particularly those where environmental conditions are hot, could consider strategies such as implementing more work/rest schedules and/or adjusting work intensity throughout a shift so to avoid excessive fatigue and thermal strain [11].

Self-pacing work intensity throughout the day is an effective approach for maintaining productivity and preventing exhaustion during a work shift. This strategy involves workers adjusting their work rate in response to a given ambient temperature, with work intensity decreasing as ambient temperature rises. For example, Brake and Bates [12] observed that most employees working in a deep underground mine in Australia were able to keep their T_c_ below 38.2°C whilst working in thermally stressful environments (31.9°C WBGT) by self-pacing their work rate, with only 7% of workers exceeding this T_c_ threshold. Importantly, allowing for planned or unplanned breaks and permitting workers to self-pace their work in hot workplaces has been shown to reduce the number of heat related illnesses [12]. However, self-monitoring and adjusting work intensity whilst working in the heat can be challenging for some workers. The development of indices such as the Thermal Work Limit requires workplace education and training for implementation [13]. Hence, many workplaces still rely on workers to regulate their work intensity based on their individual tolerance for thermal strain.

Despite the awareness of the detrimental effect of prolonged heat exposure on worker health, some workers are still required to meet quotas, driven by financial incentives for productivity, or simply obliged to complete a predetermined amount of work within their shift [13, 14]. These situations could pose a risk to the health and safety of these workers [15, 16]. To our knowledge, no studies have explored the potential disparities in thermal strain experienced or the impact on cognitive function and manual dexterity between workers who have the autonomy to self-select their work intensity and those who must adhere to a fixed-schedule workload in the heat.

Therefore, this study aimed to compare mine village workers who had the ability to self-regulate their work rate (outdoor maintenance workers) to those who could not (service workers who worked intermittently indoors and outdoors) over a 14-day swing in the heat. We hypothesised that, compared to outdoor maintenance workers, service workers would experience (i) higher step counts and heart rates (HR), (ii) elevations in T_c_ and thermal perception, (iii) exacerbated dehydration levels, (iv) heightened fatigue scores, and (v) impaired cognitive function and manual dexterity performance.

## Materials and methods

### Study design

Workers underwent assessment for cognitive function, manual dexterity, and psycho-physiological variables over the course of an 11–12 h shift in hot conditions. The outdoor workers were assessed in March 2021 where average outdoor temperature was ∼33.9ºC (range: 21.4–43.0°C), while service workers were assessed in February-March 2022 where average outdoor temperature was ∼35.4ºC (range: 23.9–46.3°C) and indoor temperature was ∼29.5°C (range: 24.0–38.1°C). Participants underwent a familiarisation session on the first day of their 14-day swing and were tested three times over the course of a swing; at the start (days 2 or 3), middle (days 7 or 8), and end (days 13 or 14). Towards the end of the swing, the number of service workers decreased because three workers left the site unexpectedly and one worker ended their workday after 5 h due to dehydration. Outdoor workers completed an 11-h shift with 60 min of meal breaks, while service workers had a 12-h shift with 90 min of meal breaks. Testing occurred pre-shift (6–7 am), mid-shift (12–1 pm), and post-shift (5–7 pm). Participants wore the same clothing for each testing session (steel cap boots, trousers, yellow-high visibility long sleeve shirt, and a hat). A food and fluid consumption diary was completed during the shift. All data was de-identified after data collection.

### Participants

Twenty-four workers volunteered for this study (Table 1), consisting of two groups: outdoor maintenance workers and service workers. Workers were recruited on the first day of their 14-day swing, in summer, and tested at the start, middle and end of the swing. All maintenance workers (n = 12; all male) had the ability to self-pace their work, which included activities such as digging, installing utilities, carrying light to heavy loads, walking, driving vehicles, and working with tools. These participants worked predominantly outdoors (∼80%). Service (cleaners) workers [male = 5, female = 7 for start and middle swing; male = 4, female = 4 for end swing) were required to clean a set number of rooms per day in the mine site village and therefore did not have the ability to regulate their work schedule. Due to workers being flown off site early and one worker not finishing their workday due to dehydration, there were only 8 service workers assessed at the end of the swing. These workers were exposed to outdoor and indoor environments intermittently (Table 2), spending approximately 15 min inside and 5 min outside, every 20 min, for ∼9 h of their shift (hence ∼135 min outdoors), with an additional 30 min of continuous outdoor exposure at the start, middle and end of the shift (∼total 90 min outdoors). Tasks completed included carrying light to heavy loads, making beds, delivering linen, cleaning bathrooms, mopping, walking, pushing trolleys, and other cleaning tasks. Participants were fly-in/fly-out (FIFO) employees who worked continuously for 14 days (swing) at mine site villages in the Pilbara region, north-west of Australia, before taking a 7-day break in Perth (all residing in a 2 h radius from Perth, Western Australia). Due to Covid-19 protocols and client approvals, participants were recruited from two different mine site villages (approx. 300 km apart) in late summer (March 2021 and February/March 2022). All participants were informed about the study’s details and requirements before providing their written informed consent. Ethics approval was granted by the Human Research Ethics Committee of the University of Western Australia (RA/4/20/6536).

**Table 1:** Demographic information for maintenance workers (n = 12) and service workers service (n = 12).

	Age (y)	Employment length (y)	Waist to hip	Height (m)	Body mass (kg)	BMI (kg/m^2^)
Maintenance Worker	46±15	2.2±2.0	0.94±0.08	1.76 (1.67–1.88)	91.0 (62.7–120)	29.8 (22.0–40.6)
Service Worker	41±17	1.2±1.8	0.87±0.10	1.70 (1.54–1.82)	78.3 (51–97)	27.0 (20.7–32.3)

Data are expressed as mean±SD or mean (range). Note that there were no significant differences between variables (p>0.05).

**Table 2:** Mean ambient conditions over the course of a shift for maintenance workers (outdoor temperature; number of testing days = 18) and service workers (both indoor and outdoor temperature; number of testing days = 25).

Occupation	Ambient temperature (˚C)	WBGT (°C WBGT)	Globe temperature (°C)	Relative humidity (%)
**Maintenance workers**	33.9±4.2	29.7±2.8	42.5±7.4	38±18
**Service workers (outdoor)**	35.4±4.5	30.4±3.7	43.9±9.1	47±21^b^
**Service workers (indoor)**	29.5±3.4^a^	23.3±2.4^ab^	28.8±3.3^ab^	46±8

Data are expressed as mean±SD.

^a^ indicates significantly different to service workers (outdoor) (p<0.05)

^b^ indicates significantly different to maintenance workers (p<0.05).

### Familiarisation session

Participants recorded their demographic and anthropometric information (Table 1) and were introduced to all the physiological equipment and perceptual scales. They then performed five trials of the manual dexterity and cognitive tasks (counting span task) to reduce any potential learning effect [17].

### Protocol

Participants were fitted with HR monitors and accelerometers, upon arrival at work. They provided a urine sample during the 30-min period prior to the start of their shift. Afterwards, they attended a ∼25 min pre-work meeting where they were assigned tasks for the day. Participants then attended morning testing (see ‘testing during the work-shift’), which was conducted outdoors in a seated position. The baseline test battery was replicated post-shift. In addition, cognitive function, manual dexterity, T_c_, HR, thermal sensation, thermal comfort and rating of perceived exertion (RPE) were measured again at midday.

### Testing during work-shift

Participants rotated through different testing stations in order to assess: 1) manual dexterity and cognitive function, 2) mood states and 3) HR, T_c_ and perceptual measures of thermal sensation, thermal comfort and RPE. The Depression, Anxiety and Stress Scale (DASS) was administered only pre-shift. Tests were performed in the same order for a given participant in all their testing sessions. Room temperature was measured during various cleaning tasks, with hourly monitoring of outdoor environmental conditions (ambient temperature, globe temperature, WBGT and relative humidity [RH]) conducted using QuesTEMP 32 (TSI Incorporated, USA; accuracy ± 0.5˚C). Wind speed was also measured at similar intervals via a digital anemometer (Model: AM-4203HA, Lutron Electronic Enterprise Co., LTD., Taiwan; accuracy 0.1 ± km/h).

#### Physiological responses

Core temperature was assessed regularly using an ingestible radio-telemetric capsule, with data transmitted to a CorTemp Data Recorder 262K device (CorTemp HQ Inc., Palmetto, USA; accuracy±0.1˚C). Heart rate was measured throughout the work-shift, on a continuous basis, using a chest-based monitor (Polar H7, Finland). Urine specific gravity was assessed using a hand-held refractometer (ATAGO Model URC-N_E_, Japan), with values classified as *‘well hydrated’* <1.010, *‘minimal dehydration’* 1.010–1.020, *‘significant dehydration’* 1.021–1.030 and ‘*serious dehydration’* >1.030 [18]. Accelerometers (Actigraph GT3X, Pensacola, USA) were worn by participants, attached to clothing near their hip, to measure the steps (activity) of each worker. This data was recorded continuously (sampling frequency 30 Hz) throughout the shift and exported to the analysis software (Actilife, version 6.13.4, Pensacola, USA).

#### Perceptual responses

Ratings of perceived exertion (6 [no exertion at all] to 20 [maximal exertion]) was measured using the Borg scale of perceived exertion [19]. Thermal comfort (0 [very comfortable] to 20 [very uncomfortable]) and thermal sensation (0 [very cold] to 20 [very hot]) were recorded using visual analogic scales ranging from white to black and green to red, respectively [20]. Higher scores for thermal comfort and thermal sensation indicated feeling less comfortable and hotter, respectively.

#### Fatigue and mental health

The DASS is a self-report scale that measures negative emotional states and is assessed using a 4-point scale (0 [never] to 3 [always]). The short-form version of the DASS, has previously been used in the Australian FIFO industry [21, 22], was administered pre-shift on all testing days. The Multidimensional Fatigue Scale, previously validated in army recruits and junior doctors [23], was used to measure physical, mental and general fatigue, as well as motivation and activity. It is scored on a scale of 1 (Yes, this is true) to 5 (No, this is not true), with higher scores representing greater levels of fatigue.

#### Manual dexterity and cognitive function

Manual dexterity (i.e. concentration, hand-eye coordination and fine motor skills) was assessed using the Purdue pegboard task (Model 32020, J.A Preston Corporation, New York) [24]. Processing efficiency and working memory capacity were assessed using a modified version of the counting span task (Inquisit Lab 6, Millisecond Software, Seattle, USA) taking ∼5 min to complete [25]. This task requires counting the green dots on a sequence of cards containing yellow and green dots and then recalling the cards in order, with set size increasing from 2 to 7. The recorded variables included counting latency, first recall latency, subsequent recall latency, number of cards counted correctly, number of cards recalled correctly and counting span [26]. Individual counting and recall latency times (ms) were aggregated across all trials with the same number of target dots, or within the relevant serial position, respectively.

### Statistical analysis

Data are expressed as mean ± standard deviation. Statistical analysis was conducted using R studio 1.4.1717. Demographic and environmental data was assessed using a one-way ANOVA. Data from all three testing days for each participant was included in the analysis (maintenance worker n = 12; service worker n = 12), however swing was not included as a factor. Linear mixed model analysis was used to compare cognitive function, T_c_, HR, RPE, thermal sensation and thermal comfort with shift and occupation (and target dots counted for counting and recall latency) included as fixed effects and participant as random effect. Fatigue, USG and manual dexterity were compared using a linear mixed model with shift, and occupation included as fixed effect and participant as a random effect, and pre and post-shift values were compared. Where appropriate, post hoc comparisons using *Tukey LSD* were conducted. Statistical significance was accepted at p<0.05. Cohen’s *d* effect sizes with ± 95% confidence intervals were calculated for primary variables (activity, HR, T_c_, USG, RPE, thermal comfort and thermal sensation) with only moderate (*0*.*50–0*.*79*) to large (*>0*.*80*) effect sizes reported.

## Results

### Environmental conditions

There were no significant differences between ambient temperature, WBGT and globe temperature for maintenance workers and service workers (outdoor) environments, however RH was significantly higher for service workers (outdoor) compared to maintenance workers (p<0.05). There was a significant difference in globe temperature and WBGT, where service workers (indoor) conditions were lower than maintenance and service workers (outdoor) conditions. Lastly, ambient temperature was significantly lower for service workers (indoor) compared to service workers (outdoor) but not maintenance workers.

### Physiological responses

#### Activity

There was a significant main effect for occupation (p<0.001; *d = 1*.*46* [0.82, 1.88]). Service workers (11282±1794) completed a significantly higher number of steps throughout the shift than maintenance workers (7774±2821).

#### Hydration

**Fig 1:**
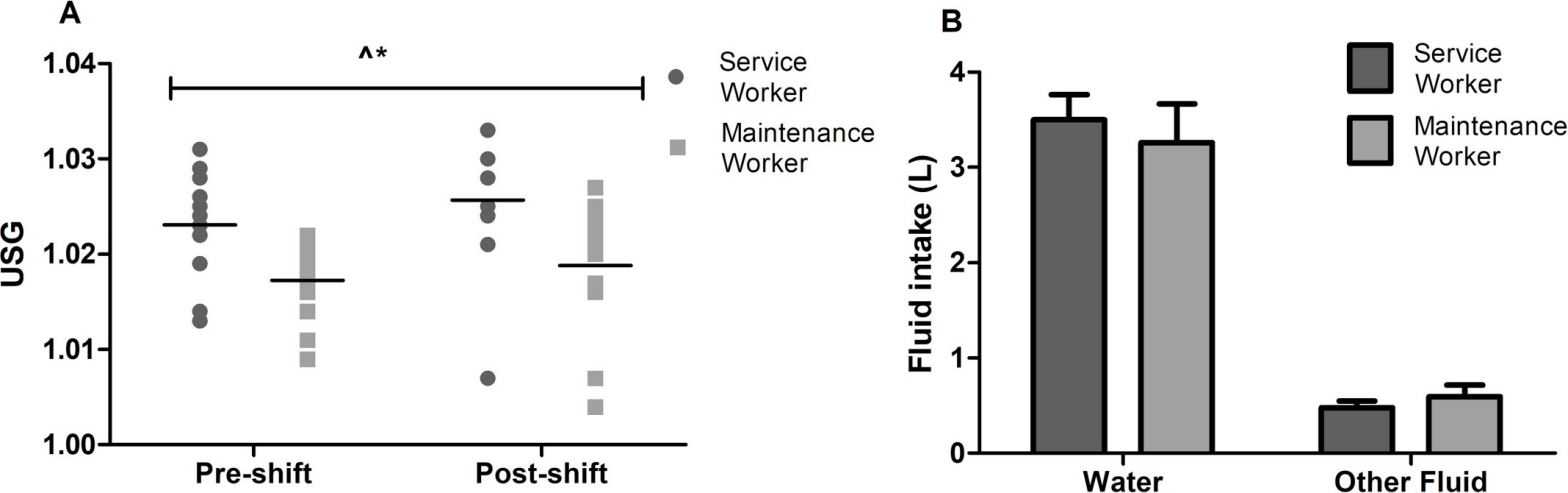
Mean urinary specific gravity scores (A) and fluid intake (B) for workers over the course of a shift (maintenance worker = 12, service worker = 12). *indicates significant main effect for shift (p<0.05); **^**indicates significant main effect for occupation (p<0.05).

There was a significant main effect of USG for both occupation (p = 0.007; *d = 0*.*92* [0.36, 1.35]) and shift (p = 0.011; Fig 1A), indicating that service workers (1.024±0.007) were more dehydrated than maintenance workers (1.018±0.006), and that workers (overall) were more dehydrated post-shift (1.022±0.008) compared to pre-shift (1.020±0.006). There were no significant interaction effects (p>0.511). Compared to maintenance workers, there was a tendency for service workers to be more dehydrated both pre (1.023±0.007 *vs* 1.017±0.005; *d = 1*.*00* [0.42, 1.42]) and post-shift (1.026±0.007 *vs* 1.019±0.008; *d = 0*.*93* [0.36, 1.35]).

Water intake did not differ between occupations (p = 0.611; Fig 1B). Mean water intake for service workers was 3.6±1.2 L and for maintenance workers was 3.3±1.5 L. Other fluid intake did not result in any main effects for occupation (p = 0.445).

#### Core temperature

**Fig 2:**
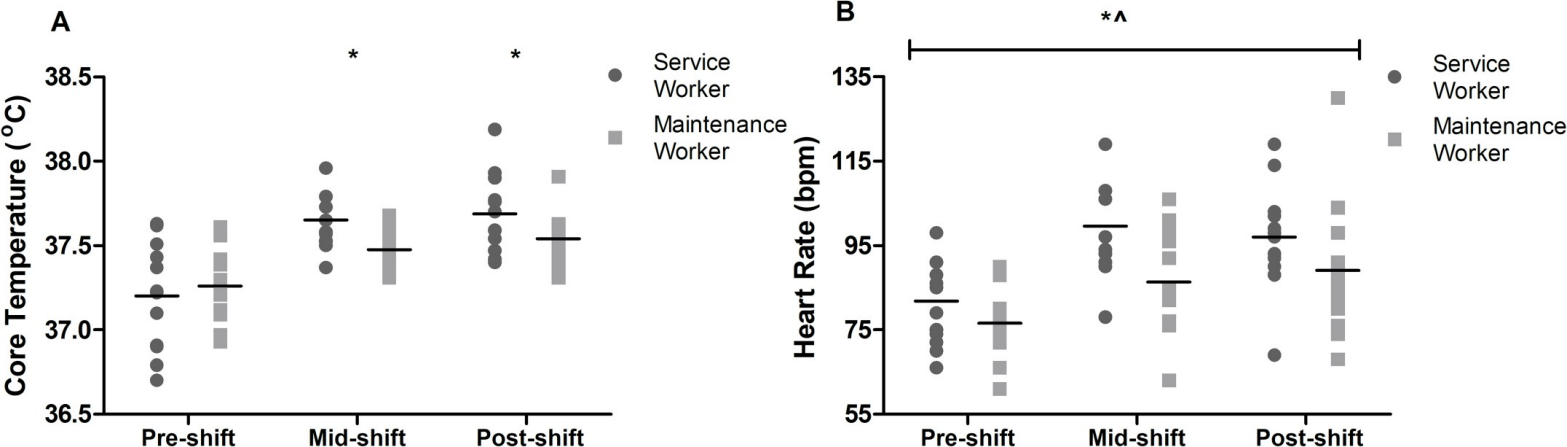
Core temperature (A; n = 22) and heart rate (B; n = 24) over the course of a shift. *indicates significant main effect for shift (p<0.05); **^**indicates significant main effect for occupation (p<0.05).

There was a significant main effect for shift (p<0.001; *d = 1*.*26–1*.*43* [0.77, 1.73]; Fig 2A), but not for occupation (p = 0.188). There was a tendency for service workers to have a higher T_c_ mid (*d = 0*.*65* [0.06, 1.13]) and post (*d = 0*.*59* [0.01, 1.08]) shift compared to maintenance workers. The interaction effect between occupation and shift was significant for T_c_ (p = 0.003), indicating that both maintenance workers (p<0.002; *d = 0*.*89*–1.11 [0.25, 1.61]) and service workers (p<0.001; *d = 1*.*56–1*.*69* [0.89, 2.12]) had a higher T_c_ mid and post-shift compared to pre-shift. Peak T_c_ for service workers was 37.82±0.22˚C and for maintenance workers 37.74±0.18˚C.

#### Heart rate

**Fig 3:**
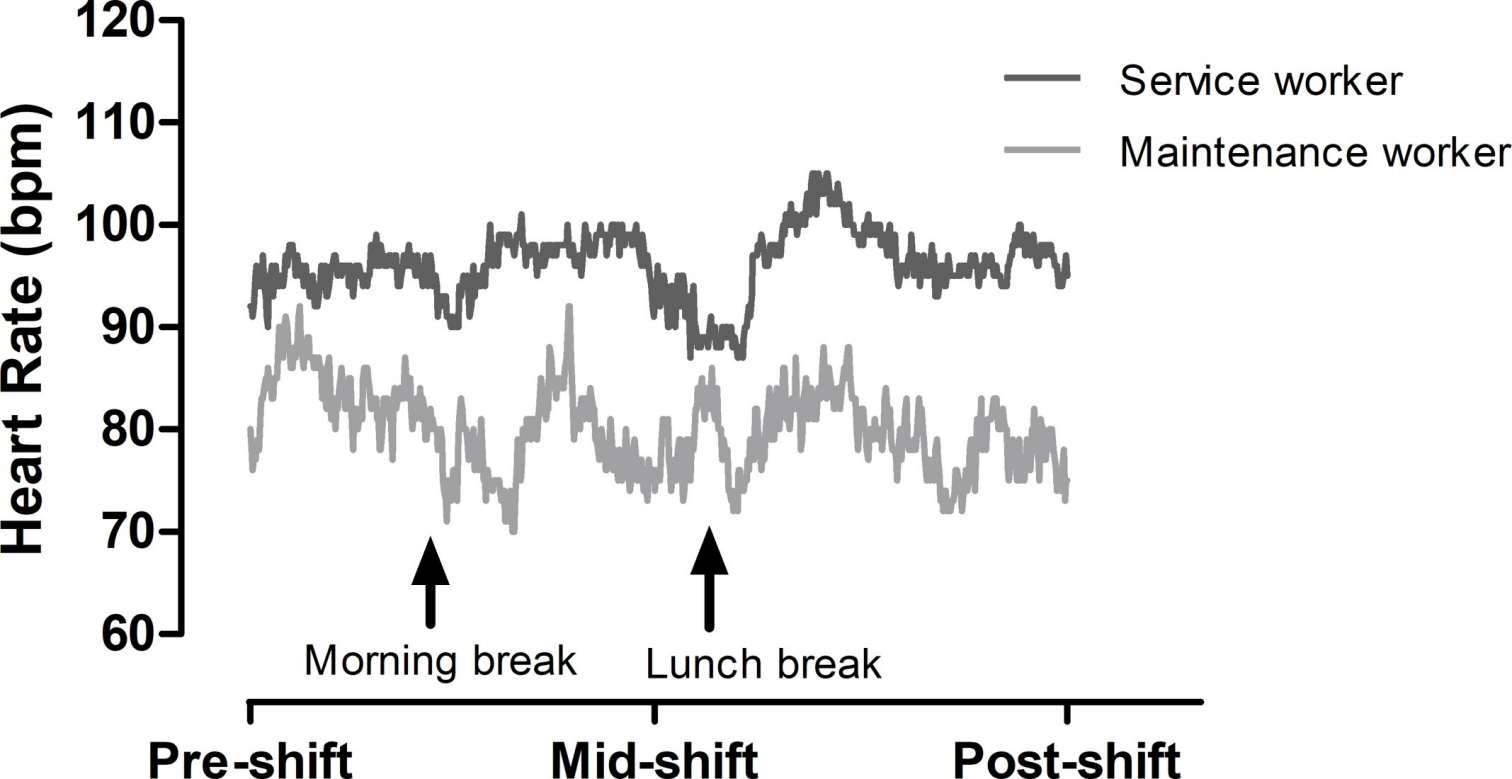
Average heart rate data over the course of a shift for maintenance (number of observations over the swing = 18) and service workers (number of observations over the swing = 30).

Significant main effects were found for occupation (p = 0.049) and shift (p<0.001; *d = 0*.*80–0*.*95* [0.39, 1.23]; Fig 2B). Service workers (93±15 bpm) had a tendency for a higher overall HR than maintenance workers (84±16 bpm; *d = 0*.*58* [0.05, 1.02]). Workers had a higher HR at mid (92±16 bpm; p<0.001) and post-shift (93±18 bpm; p<0.001) compared to pre-shift (79±11 bpm). There were no interaction effects (p>0.145). There was a tendency for HR to be higher mid and post-shift compared to pre-shift in service workers (*d = 1*.*09–1*.*35* [0.49, 1.78]) and maintenance workers (*d = 0*.*71–0*.*76* [0.18, 1.18]) independently. Peak HR for service workers was 106±14 and for maintenance workers was 100±20. Fig 3 shows an example of heart rate fluctuations of maintenance and service workers (not all participants had a continuous data export for HR).

### Perceptual responses

#### Rating of perceived exertion

**Fig 4:**
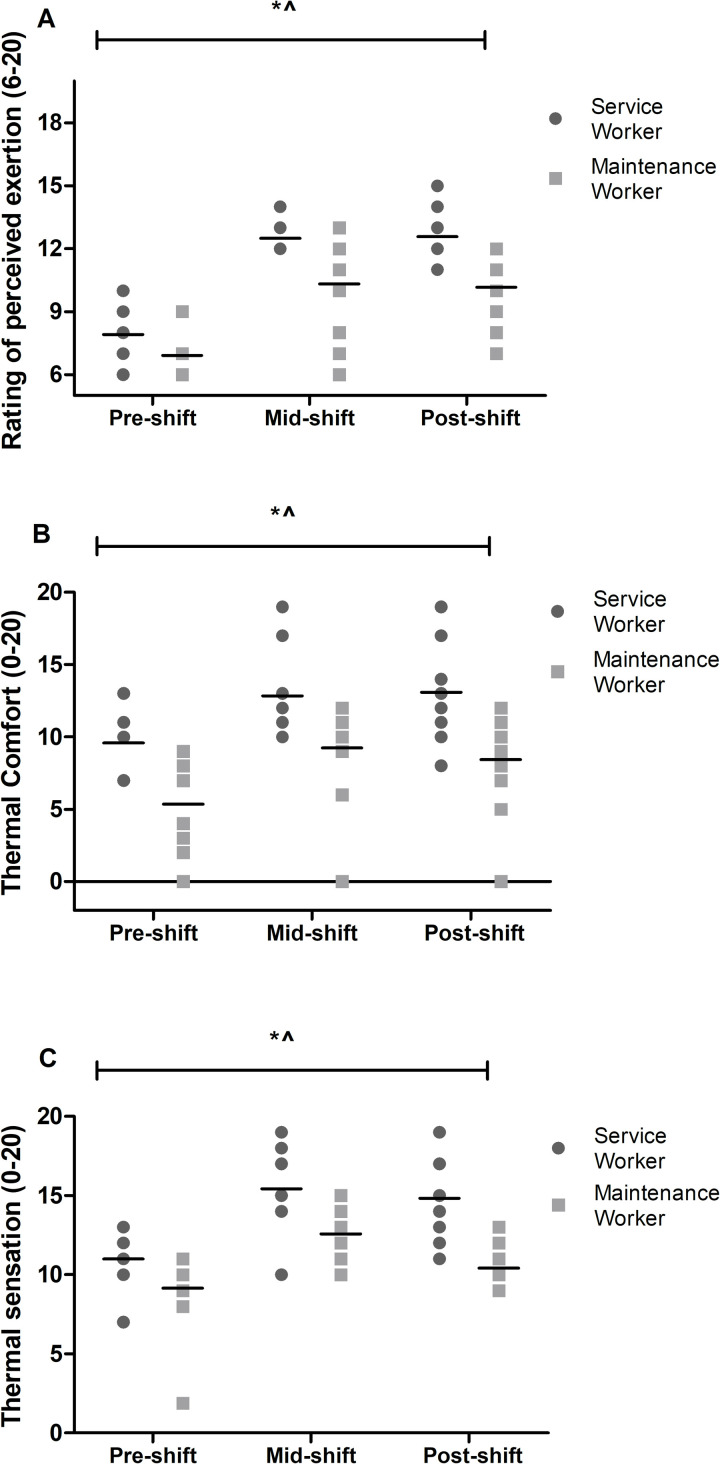
Rating of perceived exertion, thermal comfort and thermal sensation for workers over the shift (n = 24). *indicates significant main effect for shift (p<0.05); ^indicates significant main effect for occupation (p<0.05).

There was a significant main effect for both occupation (p<0.001; *d = 0*.*67* [0.13, 1.10]) and shift (p<0.001; *d = 2*.*00* [1.44, 2.25]; Fig 4A). There was no interaction effect for RPE (p = 0.095). Service workers reported higher RPE scores, mid (12±1) and post-shift (13±2) compared to maintenance workers mid (10±3; p = 0.018; *d = 0*.*87* [0.31, 1.30]) and post-shift (10±2; p = 0.002; *d = 1*.*50* [0.85, 191]) scores. Peak RPE for service workers was 13±1 and for maintenance workers was 12±3.

#### Thermal comfort

There were significant main effects for occupation (p<0.001; *d = 0*.*66* [0.12, 1.09]) and shift (p<0.001; *d = 0*.*80* [0.39, 1.09]; Fig 4B). Service workers (12±4) had a higher thermal comfort score than maintenance workers (8±5), meaning that they felt less comfortable. Workers had a higher thermal comfort score mid (11±4; p<0.001) and post-shift (11±5; p<0.001) compared to pre-shift (7±5). There were no significant interaction effect (p = 0.787). Peak thermal comfort for service workers was 15±3 and for maintenance workers 11±4.

#### Thermal sensation

Significant main effects for occupation (p<0.001; *d = 0*.*86* [0.30, 1.28]) and shift (p<0.001; *d = -0*.*57–1*.*57* [-0.86, 1.83]; Fig 4C) were found. There was a significant interaction effect between occupation and shift, where thermal sensation was higher in service workers at mid (16±2) and post-shift (15±3) compared to maintenance workers at mid (12±2; p<0.001; *d = 1*.*19* [0.59, 1.61]) and post-shift (10±3; p<0.001; *d = 1*.*55* [0.90, 1.96]). Peak thermal sensation for service workers was 17±2 and for maintenance workers was 14±2.

### Fatigue

Service workers felt greater general and mental fatigue, and were less motivated compared to maintenance workers (p<0.012; Table 3). For shift, a main effect was found for general, physical and mental fatigue, as well as reduced motivation, with workers being more fatigued post-shift than pre-shift (p<0.036). There were no main effects (p>0.205), nor interaction effects (p = 0.234) for the reduced activity domain of fatigue. There was a significant interaction between occupation and shift for general, physical and mental fatigue, and reduced motivation (p<0.05). In all domains, service workers experienced significantly greater fatigue post-shift than pre-shift, and post-shift fatigue was significantly greater in service workers compared to maintenance workers.

**Table 3:** Fatigue scores in maintenance (n = 12) and service workers (n = 12) pre- and post-shift.

	General fatigue^^c^		Physical fatigue^c^		Mental fatigue^^c^		Reduced motivation^^c^		Reduced activity	
	PRE	POST	PRE	POST	PRE	POST	PRE	POST	PRE	POST
**Maintenance workers**	8±3	8±3	7±3	6±2	7±3	7±3	6±2	7±3	7±3	7±2
**Service workers**	10±3	12±3	8±2	10±3	9±2	10±2	7v3	10v4	8±2	9±3

**^**indicates significant main effect for occupation (p<0.05)

^c^ indicates significant interaction effect between occupation and shift (p<0.05)

### Mental health

Depression did not have a significant main effect for occupation (p = 0.438). Conversely, anxiety had a main effect for occupation (p = 0.037), with services workers (6±4) reporting greater anxiety scores than maintenance workers (3±3), although both groups fell within the *“normal”* category. Lastly, stress had a significant main effect for occupation (p = 0.027), with service workers (10±6) reporting greater stress levels than maintenance workers (6±5) but again both groups fell into the *“normal”* category.

### Processing speed

#### Counting latency

Counting latency had significant main effects for shift and target dots counted (p<0.05), but not for occupation (p = 0.690). Latency was greater pre-shift compared to mid (p<0.001) and post-shift (p<0.001), and (trivially) greater for larger number of dots (p<0.001). These main effects were qualified by two significant interaction effects: occupation and target dots counted (p<0.001), and shift and target dots counted (p = 0.032). These indicated that latencies with greater target dot numbers were longer compared to smaller target dot numbers (p = 0.001). No significant differences were observed in relation to occupation and target dots counted when each occupation was counting the same number of green dots (p>0.880).

#### Counting correct responses

The number of correct responses showed a significant main effect for occupation, with service workers (47±9) counting more accurately than maintenance workers (43±11; p = 0.038). There was no main effect for shift, nor was there an interaction effect (p>0.084).

### Working memory capacity

#### Recall latency

As to be expected, first response recall latency was significantly greater than subsequent recall latency (p<0.001). Both first response latency (p = 0.007) and subsequent response latency (p = 0.042) showed a significant main effect for shift, where latencies were longer pre-shift compared to post-shift. There was no main effect of occupation for either first (p = 0.175) or subsequent response latencies (p = 0.530) and no interaction effects (p>0.651).

#### Recall correct responses

There was no significant main effects, nor interaction effect for recall correct responses (p>0.129).

#### Counting span

No significant main effects for counting span were present (p>0.413), nor were there any significant interaction effects (p>0.183).

### Manual dexterity

There was a main effect for shift (p = 0.032) for the dominant hand, but not for occupation (p = 0.064). Pre-shift (16±2) scores were significantly lower (worse) than post-shift (17±2; p = 0.002). There were no significant interaction effect (p = 0.877).

For the non-dominant hand, there were no main effects (p>0.091), nor was there a significant interaction effect (p>0.293).

## Discussion

To our knowledge, this is the first study to compare occupations in the mining industry, where maintenance workers had the ability to vary their work rate/intensity, while service workers had to maintain a set/required rate so to meet a predetermined schedule. Despite working predominantly in hot outdoor conditions (33.9±4.2°C, 38±18% RH), maintenance workers had lower HR, less dehydration and fatigue, and reported lower ratings of exertion and thermal discomfort than service workers, who were mainly working indoors (29.5±3.4°C, 48±8% RH). However, there were no significant differences between the two groups in terms of cognitive function or manual dexterity performance. The differences observed between these two groups may be related to the varying work intensities and/or exposure of service workers to the slightly higher RH (due to cyclonic conditions), which could have impacted thermoregulatory processes, in particular sweat rate and hence hydration levels.

Service workers experienced significantly greater mean dehydration (USG = 1.024±0.007; *“significant dehydration”*) than maintenance workers (USG = 1.018±0.006; *“minimal dehydration”*). Specifically, dehydration levels increased in 8 of 12 (66%) service workers during their shift, with 7 and 8 participants providing a USG value >1.030 pre-shift and post-shift, respectively. Contrastingly, no maintenance workers reported a USG >1.030, although 7 of 12 (59%) ended their shift with a greater USG value than pre-shift. There are several possible reasons for greater dehydration levels in service workers. Firstly, as service workers were unable to reduce their overall workload, prolonged periods of performing high-intensity physical activity may have encouraged sweat loss and hence dehydration (if not counterbalanced by water intake). This situation would have been further exacerbated by sustained exposure to a higher RH whilst working, which would have further increased sweat loss. Finally, inadequate pre-shift hydration among service workers, as determined by higher USG levels compared to maintenance workers, would have contributed to increasing levels of dehydration over the course of a shift if fluid intake was not encouraged. Importantly, a decrease in total blood plasma volume resulting from dehydration increases HR, decreases stroke volume, and consequently results in higher thermal strain [27].

Elevated HR can reflect physiological strain due to increased thermoregulatory demands for cutaneous blood flow as T_c_ rises [28]. If work intensity or thermal exposure do not decrease and metabolic heat production exceeds heat dissipation, this can result in heat illnesses and/or reduced productivity [28]. In the current study, service workers recorded a significantly higher average HR over the workday (∼93 bpm) compared to maintenance workers (∼84 bpm). This higher cardiovascular strain most likely reflected the combined effects of exposure to slightly higher RH and the requirement to maintain work intensity to meet a pre-established work schedule. Visual inspection of Fig 3 highlights the typical HR trend for maintenance workers, indicating that they were able to keep their HR under 90 bpm for most of the work shift, presumably by taking breaks or reducing work intensity. Conversely, for service workers the trend showed that this occupation sustained a HR above 90 bpm for most of the work shift, with HR exceeding 100 and 110 bpm for parts of the shift. Interestingly, previous literature has noted that workers who were well-educated about working in the heat and able to self-select their work intensity were able to keep their HR under 110 bpm for most of their shift duration [13]. Despite these higher HR values, it appears that the cardiovascular strain experienced by service workers in this study was not high enough to cause thermoregulatory impairment due to excessive metabolic heat production, as there were no differences in mean T_c_ between occupations and mean T_c_ did not exceed 38˚C. A limitation of this study was the lack of continuous measurement of T_c_, which may have resulted in some higher T_c_ values being missed, given that T_c_ was only monitored five times throughout a shift.

In our study, service workers reported higher levels of thermal discomfort than maintenance workers. Additionally, service workers exhibited higher levels of general, physical and mental fatigue, and lower levels of motivation compared to maintenance workers during post-shift assessments. These perceptual effects may have been caused by the inability of service workers to regulate their work intensity, as well as the effects of heat and dehydration (separately or in combination). More specifically regarding thermal discomfort, Karthick and collaegues [29] noted that workers who experienced excessive thermal discomfort in the workplace were more likely to suffer from injuries and incidents due to a lack of focus on tasks or an increased cognitive load [29]. It is therefore essential to closely monitor workers, especially during high workload periods in summer months, to ensure they maintain proper hydration levels and provide them with rest breaks in cooler environments to reduce their fatigue and discomfort levels.

While dehydration can have adverse effects on cognitive function [30], there were no significant differences in working memory or processing speed between the two occupations, despite dehydration being significantly greater in service workers. This lack of difference in cognitive function may have been due to several factors, including the fact that both groups attained a peak T_c_ that did not reach or exceed 38.5°C (a level found to impair some cognitive tasks [31]). Additionally, a practice effect due to task repetition may have benefited both occupations, and the fact that the participants were different between the two groups could also have influenced results.

### Limitations

This study is not without limitations. Firstly, despite comparable demographic details between the two occupations, workers were not the same, and individual differences (including gender) and the performance of different work tasks could have influenced results. Secondly, the amount of fluid consumed pre- or post-shift was not recorded. Monitoring fluid intake may have provided insight into the workers’ pre-shift USG values. Lastly, total body movement was not monitored, and unplanned rest breaks were not recorded for either occupation. Including real-time task analysis to track work behaviours of self-paced workers could have helped us understand more specifically whether maintenance workers paced their work intensity and/or took unplanned rest breaks as their preferred self-pacing strategy. Future studies should consider including these variables to obtain a more comprehensive understanding of the work behaviours of self-paced workers.

### Practical implications

While cognitive function and manual dexterity remained unaffected in self-paced maintenance workers and fixed schedule service workers during hot working conditions, employers should remain vigilant to the negative consequences of working in a hot environment. For self-paced workers, physically demanding labour should be scheduled during cooler parts of the day, with adequate rest breaks taken in shaded areas to facilitate cooling [8]. Encouraging workers to drink cold water during these breaks can help lower core body temperature [32] and counteract the effects of dehydration. For fixed schedule workers, employers should consider implementing extra work-rest schedules based on WBGT or thermal limit values guidelines, especially when temperatures exceed a certain threshold [33]. Additionally, employers can provide fixed schedule employees with cooling options, such as keeping air conditioning running in workspaces and/or supplying cooling modalities (cooling vests or neck cooling) that do not hinder work performance [8]. Moreover, fixed schedule workers should be informed on the benefits of ingesting cold water regularly during their shift. More research is needed to explore feasible cooling modalities that can effectively reduce thermal perception, perceived fatigue, and heart rate in the field.

## Conclusion

This study is the first in the mining industry to directly compare the physiological, perceptual and cognitive responses between workers who could regulate their work intensity (maintenance workers) and those who worked to a fixed schedule to meet work requirements (service workers). Service workers had worse/elevated heart rates, dehydration, fatigue and measures of exertion and thermal discomfort, while there was no significant difference in T_c_, cognitive function, and manual dexterity performance between the two occupations. Policies regarding occupational heat stress and exposure in the Australian mining industry need refinement in order to protect workers (especially for those working to a fixed schedule) from the risks of exertional heat illness and injury.

## Supporting information

S1 File Demographics. (XLSX)

S2 File Water intake, activity & DASS. (XLSX)

S3 File Physiology, perceptual & cognitive. (XLSX)

S4 File Fatigue, USG & manual dexterity. (XLSX)

S5 File Correct response. (XLSX)

S6 File Counting latency. (XLSX)

S7 File Recall latency. (XLSX)

S8 File Average heart rate over shift. (XLSX)

S9 File Environmental data. (XLSX)
